# Partial Laryngectomy for Thyroid Cancer: A Single Institution Case Series With Operative Techniques

**DOI:** 10.1002/hed.28246

**Published:** 2025-08-01

**Authors:** Ella E. Hawes, Victoria E. Banuchi, Anastasios Maniakas, Jennifer R. Wang, Paul H. Graham, Julia D. Diersing, Naifa L. Busaidy, Maria E. Cabanillas, Ramona Dadu, Sarah Hamidi, Priyanka Iyer, Steven G. Waguespack, Salmaan Ahmed, Sahar Alizada, Alexandra Belcastro, Ranim Alsharif, Mimi I. Hu, Mark E. Zafereo

**Affiliations:** ^1^ Department of Head and Neck Surgery The University of Texas MD Anderson Cancer Center Houston Texas USA; ^2^ Department of Surgical Oncology The University of Texas MD Anderson Cancer Center Houston Texas USA; ^3^ Department of Endocrine Neoplasia and Hormonal Disorders The University of Texas MD Anderson Cancer Center Houston Texas USA; ^4^ Department of Diagnostic Radiology The University of Texas MD Anderson Cancer Center Houston Texas USA

**Keywords:** larynx, partial laryngectomy, surgery, thyroid cancer

## Abstract

**Background:**

Partial laryngectomy has been well described as a management option for T1–3 laryngeal squamous cell cancers. However, there are a few case reports of partial laryngeal surgery for locally advanced thyroid carcinoma.

**Methods:**

This is a case series of five patients with thyroid carcinoma (one primary and four recurrent) with laryngeal involvement who underwent partial laryngectomy.

**Results:**

All patients had complete surgical resection without perioperative complications or postoperative speech or swallowing deficits, and none required a feeding tube or tracheostomy tube. No patients had postoperative radiation therapy or started systemic therapy within 2 years of surgery, while two patients had postoperative radioactive iodine. With a mean 26‐month follow‐up, only one patient had recurrence involving the larynx (cricoid cartilage recurrence at 26 months).

**Conclusion:**

Partial laryngectomy should be considered for patients with thyroid cancer with limited laryngeal involvement.

## 1 Introduction

Approximately 10% of thyroid cancer is locally advanced, although laryngeal invasion is very rare [1]. Laryngeal invasion occurs by direct extension through the thyroid lamina, infiltration through the cricothyroid membrane, or posterior invasion through the pyriform sinus [2]. While total laryngectomy is rarely required for patients with advanced thyroid cancer, neck breathing and loss of normal laryngeal voice negatively impact quality of life [3]. Partial laryngectomy is well described in the treatment of patients with intralaryngeal squamous cell carcinoma, but has only been reported for advanced thyroid cancer in a few case reports [4, 5, 6, 7, 8, 9, 10]. The pattern of potential laryngeal invasion in thyroid cancer differs from that of laryngeal cancer, as it typically progresses from the outer side of the cartilage. This often allows for potential preservation of the mucosa of the larynx and pharynx during surgery, with early functional recovery postoperatively. Partial laryngectomy can generally be performed if less than 50% of the laryngeal framework and cricoid are involved, and this procedure has a clear functional benefit over total laryngectomy [2]. Contraindications to partial laryngectomy (and potential indications for total laryngectomy) include significant laryngeal mucosa or pharyngeal involvement, contralateral recurrent laryngeal nerve paralysis, and greater than 50% of laryngeal or cricoid invasion [2]. Herein, we present five cases of locally advanced thyroid cancer treated with partial laryngectomy, the largest such series to date. After institutional review board approval (PA14‐1082) and a retrospective review of all patients undergoing surgery for thyroid cancer at The University of Texas MD Anderson Cancer Center between 2018 and 2024, we identified five patients who had undergone partial laryngectomy.

## 2 Case Reports

### 2.1 Case 1

**FIGURE 1:**
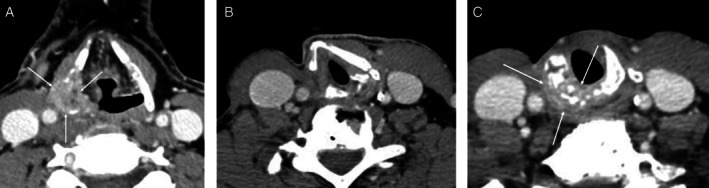
(A) Preoperative CT neck with contrast axial cut demonstrating a 2.6 cm tumor (arrow) involving the right thyroid cartilage and extending into the paraglottic space. (B) CT neck with contrast axial cut 9 months after surgery demonstrating right thyroid cartilage defect, without evidence of disease. (C) CT neck with contrast 2 years after surgery demonstrating disease recurrence in the right cricoid cartilage (arrow).

A 65‐year‐old immunosuppressed male with myelodysplastic syndrome status post stem cell transplant presented to our institution with recurrent poorly differentiated thyroid carcinoma following two previous surgeries. CT neck with contrast demonstrated disease involving the right thyroid hemicartilage and extending intralaryngeally to abut the right pyriform sinus without invasion of the pyriform sinus mucosa (Figure 1A). He was asymptomatic with bilateral vocal fold mobility. Partial right laryngectomy, including en bloc excision of the complete right thyroid lamina, was performed, after which an inferiorly pedicled right sternocleidomastoid muscle was rotated over the exposed laryngeal mucosa. There were no surgical complications, and postoperative radiation therapy was not recommended. Follow‐up CT neck imaging 1 year later demonstrated no evidence of disease (Figure 1B). At 26 months following surgery, the patient presented with asymptomatic disease within the right cricoid cartilage (Figure 1C), in addition to progression of distant disease. He started lenvatinib and had a partial response in both the cricoid metastasis and lung metastases at last follow‐up, 34 months postoperatively. He died at 50 months of follow‐up due to complications associated with his myelodysplastic syndrome.

### 2.2 Case 2

**FIGURE 2:**
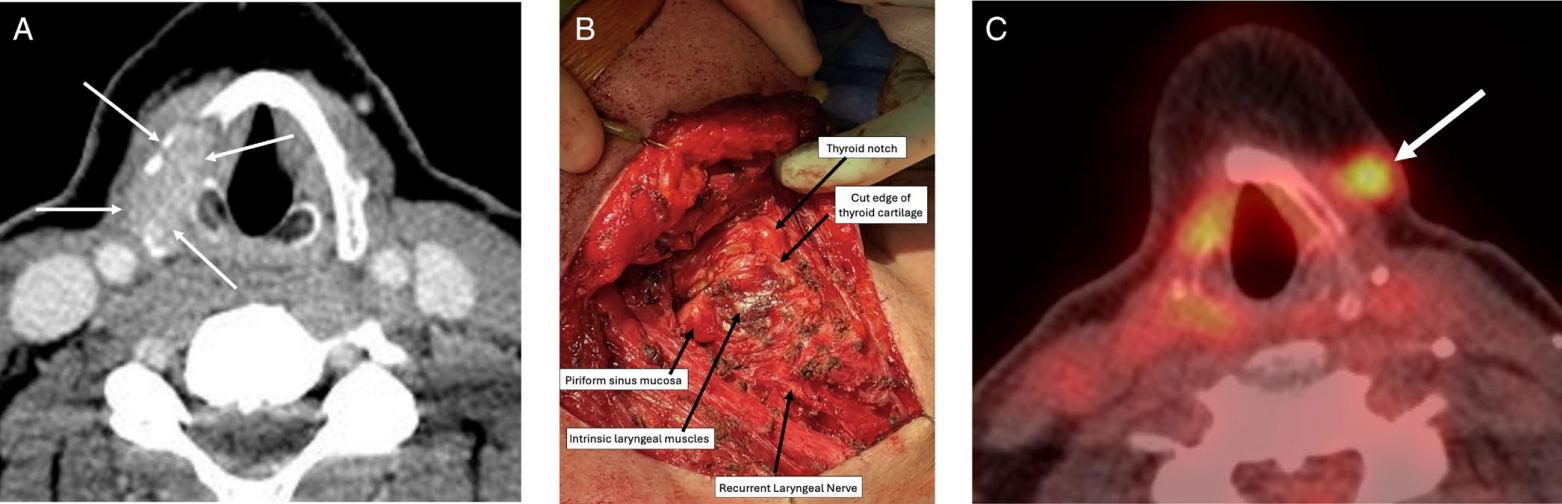
(A) Preoperative CT neck with contrast showing a 2.4 cm tumor (arrow) involving the right thyroid cartilage and extending into the paraglottic space. (B) Intraoperative photograph after right partial laryngectomy demonstrating laryngeal defect, with intrinsic muscles of the larynx, pharyngeal sinus mucosa, and intact recurrent laryngeal nerve. (C) Postoperative FDG PET CT scan 12 months after surgery showing the right thyroid cartilage defect, without evidence of laryngeal disease, but with a new 0.7 cm FDG avid focus of soft tissue disease superficial to the contralateral (left) sternohyoid muscle.

A 62‐year‐old male with a history of four previous surgeries and radioactive iodine (RAI) presented with recurrent, metastatic oncocytic thyroid cancer. He had three progressive, fluorodeoxyglucose (FDG)‐avid right neck soft tissue foci of disease, including disease in right Level VI, disease involving the right thyroid cartilage (Figure 2A), and right retropharyngeal disease. He was asymptomatic from the recurrent disease, with normal vocal cord mobility. Given the growth of the neck disease with stable hilar and lung metastases, we recommended surgical resection with palliative intent to delay the initiation of systemic therapy. A partial right laryngectomy with an en bloc excision of the complete right thyroid lamina was performed, with an inferiorly pedicled right sternocleidomastoid muscle rotating over the exposed laryngopharyngeal mucosa. The proximal right recurrent laryngeal nerve maintained stimulation after the dissection. The patient recovered well from surgery, and postoperative imaging 12 months after surgery was without evidence of disease in the right neck/larynx (Figure 2C), although the patient had a new sub‐centimeter contralateral neck disease and new distant metastases.

### 2.3 Case 3

**FIGURE 3:**
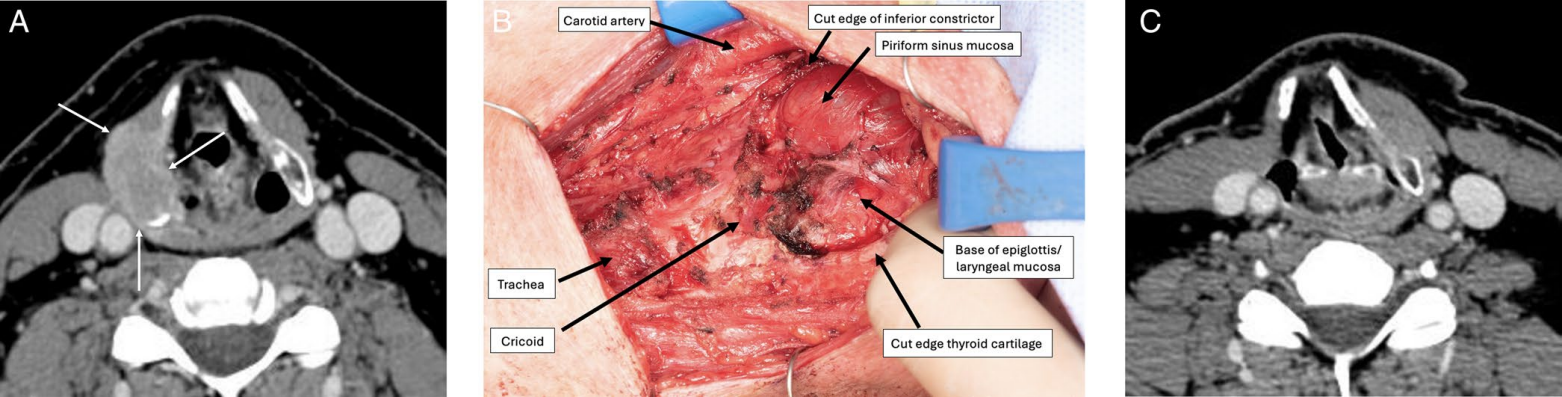
(A) CT neck with contrast delineating 2.8 cm recurrent papillary thyroid carcinoma (arrow) invading the right thyroid cartilage and extending into the paraglottic fat. (B) Intraoperative photograph following extended right hemithyroid cartilage resection (right and partial left), demonstrating surgical defect, with ballooning pharyngeal mucosa and base of epiglottis with associated mucosa. (C) CT neck with contrast 6 months after surgery demonstrating right thyroid cartilage defect and dilated right pyriform sinus, without evidence of disease.

A 57‐year‐old male presented with a 2.8 cm recurrent papillary thyroid cancer (PTC) invading the right thyroid cartilage (Figure 3A) after previous surgery (total thyroidectomy with thyroid cartilage shaving and lateral neck dissection) and RAI. Partial right laryngectomy with en bloc excision of the complete right thyroid lamina was performed (Figure 3B), rotating the ipsilateral sternohyoid muscle over the exposed laryngopharyngeal mucosa. Postoperatively, there were no complications, and normal vocal cord mobility was observed. The patient received additional RAI therapy and remained without evidence of locoregional disease 50 months after surgery.

### 2.4 Case 4

**FIGURE 4:**
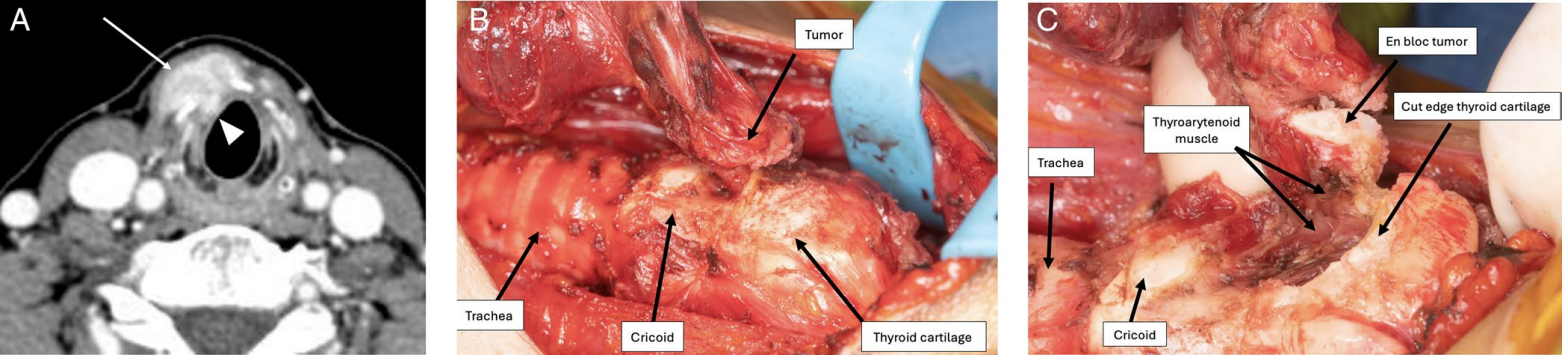
(A) Preoperative axial CT with contrast demonstrating 2.7 cm thyroid isthmus papillary thyroid cancer (arrow) infiltrating through the cricothyroid membrane but without tracheal invasion. (B) Intraoperative photograph of papillary thyroid carcinoma invading through cricothyroid membrane. (C) Intraoperative photograph demonstrating resection of the tumor en bloc with the right inferior thyroid cartilage and the right thyroarytenoid muscle on the deep margin of the tumor.

A 72‐year‐old female presented with a 2.2 cm PTC arising from the isthmus that invaded through the right cricothyroid membrane and tracked under the thyroid cartilage (Figure 4A). She underwent right partial laryngectomy, removing en bloc the right cricothyroid muscle, right inferior thyroid lamina, and right thyroarytenoid muscle (Figure 4B,C). The sternohyoid muscles were reapproximated over the defect in the midline. The patient had mild temporary hoarseness with normal vocal cord mobility and no other complications. She received 107 mci RAI 2 months after surgery, and imaging 10 months postoperatively was without evidence of disease recurrence.

### 2.5 Case 5

**FIGURE 5:**
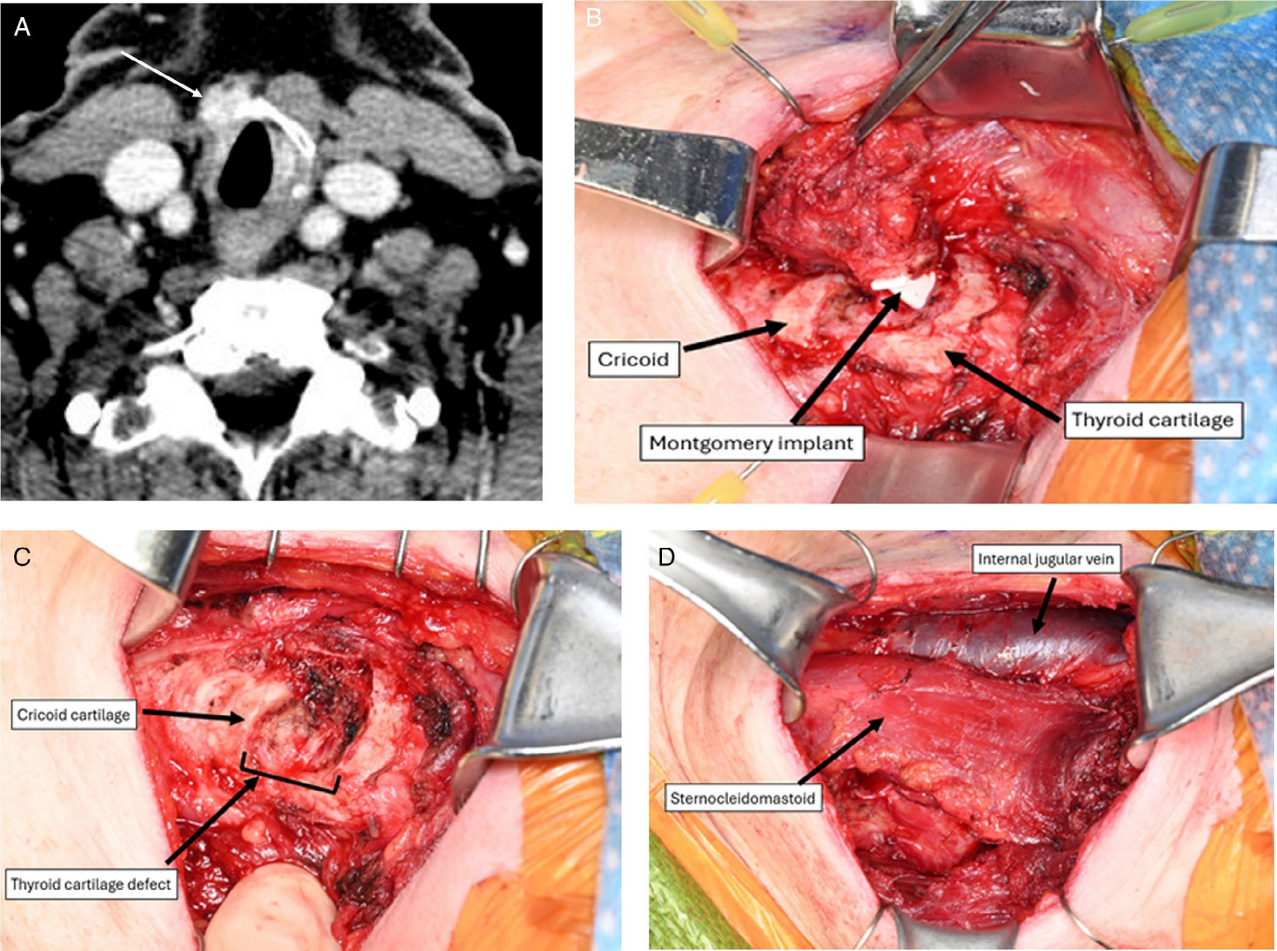
(A) Preoperative CT with contrast demonstrating a 1.7 cm nodule (arrow) in the right prelaryngeal area invading the anterior right thyroid cartilage. (B) Intraoperative photograph demonstrating right partial laryngectomy, en bloc removal of right cricothyroid muscle, right inferior thyroid cartilage, right thyroarytenoid muscle, and Montgomery implant. (C) Intraoperative photograph demonstrating laryngeal defect following en bloc tumor resection. (D) Intraoperative photograph demonstrating an inferiorly based right sternocleidomastoid muscle flap rotated to cover the laryngeal defect.

A 66‐year‐old female with a history of PTC, two previous surgeries, RAI, and thyroplasty for right vocal fold paralysis presented with recurrent PTC invading the right cricothyroid membrane and involving the inferior right thyroid cartilage (Figure 5A). She underwent a right partial laryngectomy, removing en bloc the right cricothyroid muscle, right inferior thyroid lamina, right thyroarytenoid muscle, and the Montgomery implant (Figure 5B,C). An inferiorly based sternocleidomastoid flap was rotated and sutured to the sternohyoid and sternothyroid muscles to cover the defect (Figure 5D). The patient's postoperative imaging 6 months after surgery showed no evidence of disease.

## 3 Discussion

We report the largest series of patients (five) to date undergoing partial laryngectomy for thyroid cancer with laryngeal cartilage invasion. Previously, nine other cases of partial laryngectomy for thyroid cancer have been reported across seven studies [4, 5, 6, 7, 8, 9, 10], with most (six) of these patients undergoing at least temporary tracheostomy tube placement [4, 5, 6, 7].

In the current study, four patients had near complete resection of the unilateral thyroid ala (hemicartilage), while one patient had resection of the inferior aspect (< 50%) of the thyroid ala. None of the patients in this series had cricoid cartilage involvement. Cricoid cartilage involvement > 50% is a contraindication to partial laryngeal surgery, while resection of small focal segments of the anterolateral hemicricoid cartilage can generally be performed without cartilaginous reconstruction. Resection of < 50% of the cricoid can, in some unique circumstances, be reconstructed by rotating and suturing thyroid cartilage or tracheal cartilage into the defect. For example, reconstruction of a partial laryngectomy with involvement of the lower hemithyroid cartilage and < 50% cricoid involvement has been previously described, essentially with suturing of the upper thyroid cartilage to the residual cricoid cartilage [9]. All five patients in the current series had resection of some associated paraglottic soft tissue and/or intrinsic laryngeal musculature, but without endolaryngeal mucosal defect, such that none of these patients required temporary feeding or tracheostomy tubes. Median hospital stay was 1 day (range 1–3 days), and no patients had speech or swallowing deficits related to the partial laryngeal surgery, including no new vocal cord paralyses.

These cases were performed with cartilage resection while sparing the laryngopharyngeal mucosa. Cartilage resection was performed with an electric drill with a 2–3 mm cutting bur, incising the cartilage in a broad plane away from the tumor. A freer was then used to separate the cartilage from the paraglottic space while lifting the hemicartilage flap from medial to lateral. Dissection continued until the tumor was encountered, at which point some involved muscle and/or paraglottic space fat was variably excised en bloc with the tumor. Care was taken to avoid breaching the laryngopharyngeal mucosa.

While the laryngopharyngeal mucosa was not breached in these patients, they had been prepared for the possibility of a mucosal breach, which would have necessitated NPO status with a temporary feeding tube and possibly tracheostomy tube placement. In all five cases, the sternohyoid or sternocleidomastoid muscle was rotated to cover the laryngopharyngeal mucosa. Patients were allowed to eat immediately after surgery, had a 1–3 day hospital stay, and were discharged with a small suction drain. No patients had postoperative radiation therapy or started systemic therapy within 2 years of the partial laryngeal surgery, while two patients had RAI postoperatively. With a mean 26‐month follow‐up, three patients remained without structural evidence of disease. One patient remained under active surveillance with sub‐centimeter contralateral neck and distant recurrence, while another had cricoid cartilage and distant recurrence and ultimately died with complications of myelodysplastic syndrome over 4 years after partial laryngeal surgery.

Select patients with thyroid cancer invading the thyroid cartilage and/or cricothyroid membrane can be surgically managed with partial laryngectomy with preservation of speech and swallowing function, immediate resumption of oral feeding, and avoidance of tracheostomy. Partial laryngectomy can be performed in select patients with limited thyroid cancer involvement of the thyroid and cricoid cartilages and with limited disease involvement of the paraglottic space and laryngopharyngeal mucosa. While patients in this series had quick recovery from surgery and minimal postoperative morbidity, patients undergoing this procedure should be generally prepared for the possibility of a temporary feeding tube and/or tracheostomy tube if the laryngopharyngeal mucosa is breached. In addition, surgeons performing partial laryngeal surgery for thyroid cancer should be prepared to rotate local muscle flaps (e.g., strap or sternocleidomastoid muscle) to bolster thin or breached laryngopharyngeal mucosa.

## Conflicts of Interest

Mark E. Zafereo reports research funding/grants to MD Anderson Cancer Center from Exelixis, Eli Lilly, and Merck. Anastasios Maniakas reports research funding from JAZZ Pharmaceuticals and Thryv Therapeutics Inc. Maria E. Cabanillas has received consulting fees from Bayer, Exelixis, Lilly, Novartis, and research funding from Merck, Genentech, Eisai, Exelixis. Naifa L. Busaidy reports research funding from Eisai and personal consulting fees from Eisai and Eli Lilly. Ramona Dadu reports research funding from Eisai, Merck, Exelixis, and AstraZeneca, and personal fees from Bayer and Exelixis. The other authors declare no conflicts of interest.

## Data Availability

The data that support the findings of this study are available on request from the corresponding author. The data are not publicly available due to privacy or ethical restrictions.
